# Heat Shock Protein 70 Gene Single Nucleotide Polymorphism and Diabetic Foot Ulcer. Is There Any Relationship?

**DOI:** 10.3390/jcm7080187

**Published:** 2018-07-27

**Authors:** Mohammad Zubair, Jamal Ahmad

**Affiliations:** 1 Department of Medical Microbiology, Faculty of Medicine, University of Tabuk, Tabuk 71491, Saudi Arabia; 2 Rajiv Gandhi Centre for Diabetes and Endocrinology, Faculty of Medicine, J.N. Medical College, Aligarh Muslim University, Aligarh 202002, India; jamalahmad11@rediffmail.com

**Keywords:** DFU, SNP, HSP70-Hom, homozygous, heterozygous, frequency

## Abstract

**Objective:** The study aims to investigate the potential role of C2437T (Met493Thr) single nucleotide polymorphism (SNP) of the heat shock protein (HSP) 70 in diabetic foot ulcer patients. **Methods:** In this prospective cohort study, SNP of the HSP70 hom gene, also called HSPA1L, was studied among diabetic patients with an ulcer (Group A: *n* = 50), diabetic patients without an ulcer (Group B: *n* = 50), and healthy subjects (Group C: *n* = 50). **Results:** There was a higher frequency of T/T genotype in group A (76%) as compared to group B (44%) and group C (14%). Moreover, the frequency of T allele was 7.3% in group A, 5.5% in group B, and 3.9% in group C. C allele frequency was 2.6%, 4.4%, and 6.1% in group A, group B, and group C, respectively. In group A, the odds ratio and risk ratio were 19-fold and 5-fold, respectively, for the HSP70 hom T/T homozygous gene compared to B (OR 19.45; RR 5.42; *X*^2^ 38.8, *p* < 0.0001). Moreover, 4-fold and 1.75-fold ratios have been compared with group C (OR 4.03; RR 1.72; *X*^2^ 10.6, *p* < 0.001). No significant difference in genotype was observed in group B and group C. **Conclusions:** There is a significant and positive association of hspHSP70 hom polymorphism restricted to T allele in homozygous and heterozygous states among diabetic foot ulcer (DFU) patients.

## 1. Introduction

The presence of type 2 diabetes mellitus (T2DM) is a health concern that adversely impacts the quality of life of affected individuals [1]. By the year 2030, it is expected that the prevalence of individuals afflicted with diabetes mellitus (DM) will expand to 366–440 million of the global population [2]. A wide variety of treatments is utilized for DM, such as pharmacotherapeutics and maintaining a healthy diet and lifestyle [3]. However, meeting the goals of these treatments remains widely unachieved by a majority of DM patients. Therefore, it is clear that the risks posed by DM by way of mortality and morbidity presents as a globally unsolved complication in health sectors today.

As estimated in a previous study, diabetic complications in the form of DFU severely affect 15% of DM patients, leading to infections; consequently, tissue damage occurs, due to which amputation may have to be carried out [4]. In fact, foot complications present as a predominant issue in individuals afflicted with T2DM and are inclusive of gangrene [5], ulcerations, and neuropathy [4]. Accordingly, there are significant health and economic burdens faced by diabetic patients suffering from foot complications of which the majority were DFU-related [6]. The aforementioned study reported that the prevalence of DFU was significantly greater among the male diabetic patients than female ones. Moreover, the risk associated with the development of this condition increased with the duration of DM and the age of the patient. Furthermore, DFU is associated with cellular activity changes, growth factor activation and microcirculation, due to which the process of wound healing is greatly impeded [4]. Additionally, a study noted that DFU is closely associated with reduced levels of adiponectin in addition to an enhanced level of plasma with respect to hs-CRP, TNF-α, and IL-6 [7]. 

With regard to expediting the wound healing process in DFU patients, a discussion of HSPs is extremely relevant. Although HSPs are normally present at low concentrations within the body, they are synthesized at high concentrations under conditions of high stress [8]. Specifically, 70 kd HSPs possess the ability to bond with chains of polypeptides, through which the cell entity is maintained and incorrect folding of intracellular proteins is impeded; thereby averting potential denaturation of proteins [9]. The presence of elevated plasma levels pertaining to HSP-47 in addition to HSP70 was noted to be greater in DFU-afflicted diabetics as compared to diabetics, who did not suffer from DFU [8]. The HSP70 is the most widely distributed group of HSPs of 70 kDa. All its family members contain conserved amino-terminal ATPase domain of 45 kDa with C-terminal domain, which is classified further into C-terminal subdomain of 10 kDa and substrate binding domain of ~15 kDa. Members of the family contain an extension of short N-terminal/C-terminal at the appropriate cellular compartment for targeting to, or retention in. The HSPs which are extracellular play a key role in induction of cellular immune response. To induce pro-inflammatory cytokine secretion from monocytes, the uncomplexed HSP binds to the receptors on antigen-presenting cells (APC) of HSP [10,11]. The HSPs may act as carrier molecules for immunogenic peptides that are presented on APC to cytotoxic T cells or act as activatory molecules for the innate immune system [12], and also act as a recognition structure for natural killer (NK) cells [13]. Three members of the HSP70 gene family are located between complement (~150 kb) and TNF genes (~300 kb) in the MHC class III region in humans [14,15]. HSPs were classified as HSP70-1, HSP70-2, and HSP70-hom, while HSP70-1 and HSP70-2 share an identical protein product, and act as a major heat-inducible HSP70. The HSP 70 family were encoded by HSP70-hom gene [16], which is not heat inducible. The two coding variations have been identified within HSP70-hom [15]. The first one is T to C transition at nucleotide 2437 (resulting in a Met-493-Thr variation), which lies within an NcoI restriction site [16]. The second coding polymorphism is G to A transition at nucleotide 2763 of HSP70-hom [17], located at 10 kDa domain implicated in the regulation of substrate binding resulting in a glutamic acid to lysine alteration at position 602 in the C-terminal domain of HSP70-hom (Glu602Lys variation). The polymorphism of HSP70 is not significantly associated with surgical outcomes in patients afflicted with DFU [18]. However, there is a lack of research surrounding the polymorphism of HSP70-hom in DFU. Therefore, this study has aimed to investigate the single nucleotide polymorphisms (SNP) of the HSP70-hom gene and its association with DFU and ulcer severity.

## 2. Materials and Methods

This study was hospital-based and conducted between September 2013 and February 2016 at the Rajiv Gandhi Centre for Diabetes and Endocrinology (RGCDE), Jawaharlal Nehru Medical College Hospital, Aligarh Muslim University, Aligarh, INDIA and the Department of Medical Microbiology, Faculty of Medicine, University of Tabuk, Tabuk, Kingdom of Saudi Arabia. The study recruited 150 individuals, who were classified into three groups of interest. Group A was comprised of diabetics presenting with foot ulcers, who were fifty in number. Group B was comprised of diabetic patients who did not present with any foot infection or ulceration; they were fifty in number as well. This group was comprised of individuals who had presented to the clinic for admission pertaining to other reasons. Lastly, Group C was categorized as the control group and was composed of 50 healthy volunteers. Individuals afflicted with recent venous thromboembolism were not included. Additionally, those individuals were excluded who were afflicted with conditions other than foot-related complications, critical liver and renal failure, autoimmune diseases, cancer, and rheumatoid arthritis and those undergoing treatment through the use of anti-inflammatory medication. The study was carried out in accordance with principles of the Declaration of Helsinki as revised in 2001, and subsequent consent of the patients was taken to participate in the study.

A former study by the researcher served as the basis for this study’s clinical evaluation [7]. A matching process was carried out, whereby each participant belonging to group A was made to correspond to similar participants in groups B and C on the basis of sex and age factors (±3 years). Consequently, the study investigated the participants on the basis of DNA. This part of the study was performed in the Medical Microbiology laboratories within University of Tabuk in Saudi Arabia. Blood samples were obtained from each of the participants. Additionally, the level of HSP70 plasma concentrations within these samples was determined using vitro ELISA (Bmassay Bio Medical Assay, Beijing, China). The aforementioned kit presented a range of detection between 100 pg/mL and 10,000 pg/mL. Additionally, it had a sensitivity of less than 90 pg/mL. The DNA was additionally isolated from the peripheral blood cells through the use of a PureLink Pro96 genomic DNA purification kit (Life Technologies, Carlsbad, CA, USA).

Following DNA isolation, a polymerase chain reaction (PCR) technique was used to amplify the DNA by using BioRad thermocycler (Germany). This technique is significant because it aided in detecting the polymorphisms of the HSP70-hom gene. In order to conduct the PCRs, a PCR master mix was used, which was obtained from Fermentas. This mix was composed of 2 mm of magnesium chloride; 0.05 U/mL of taq polymerase in reaction buffer; and 0.4 mm of dTTP, dGTP, dCTP, and dATP (dnTPs). In this regard, the sense primer used was 5′ GAT CCA GGT GTA TGA GGG 3′. Additionally, the antisense primer was 5′-GTA ACT TAG ATT CAG GTC TGG-3′. As per the cycling condition of the PCR technique, the primers were firstly incubated for a time duration of five minutes under 94 °C. The same temperature was maintained under which 30 denaturation cycles were conducted for a time duration of thirty seconds. Based on the composition and length of the primers, 55 °C was chosen as the annealing temperature for a time duration of twenty seconds. Furthermore, one-minute primer extension was carried out under 72 °C. The same temperature was maintained for performing a last extension of the primers, for a time duration between ten to twelve minutes.

An agarose gel with a concentration of 1.5% was used in order to initially visualize a segment of the PCR product. Consequently, the product remainder was digested through the use of restriction endonucleases. This digestion was carried out under a temperature of 37 °C between 130 to 150 min in a 1× Onephor buffer. EtBr was then used for staining and a UV trans-linker was used in order to provide fluorescence. The Ncol site was absent with respect to the correspondence of the HSP70-hom C/C allele with an un-cleaved 706 bp product. Furthermore, the Ncol site was present with respect to the correspondence of the HSP70-hom T/T allele with 550 and 155 bp products.

The obtained data was analyzed, statistically. The usage of numbers and percentages was implemented with respect to qualitative variable expression and mean ± standard deviation was used in order to express the quantitative variables. Non-parametric U-Mann-Whitney or Student T-tests were applied in order to calculate the variation between the participant groups. Additionally, variable normality was analyzed through the use of the Shapiro-Wilk test. Under the condition that normality was ascertained, risk and odds ratios having a confidence interval (CI) of 95% were stated with respect to the independent variables that were linked to the outcome variable. The risk ratio corresponded to the probability and the odds ratio corresponded to association strength. The outcome variable was ulcer severity. These ratios were additionally used in order to investigate the link between ulcer severity and the polymorphism of the HSP70-hom gene with respect to the three groups. Additionally, the use of a Chi-square test was implemented for evaluating the relationship between SNP of the HSP70-hom gene and every clinical variable. The Chi test was used due to its capability for independently predicting the development of foot ulcers at *p* < 0.05, where significance was considered for *p*-values lower than 0.05. The analysis was completely conducted through the use of SPSS 19.0 software.

**Ethical Clearance**: This study was approved by the Bio-Ethical Committee (BEC) of the Faculty of Medicine, Aligarh Muslim University, Aligarh on August 2013, registered under the Drug Controller General of India (DCGI), Government of India under registration Number: ECR/419/Inst/UP/2013 issued under Rule 122DD.

## 3. Results

The demographics pertaining to the 150 study participants across the three groups A, B, and C have been amply illustrated in Table 1. The trend of higher male diabetics was seen in these groups, whereby 66% of the participants of group A were male, 62% in group B were male, and lastly 58% in group C were male. When comparing between groups A and B, it was seen that 16 participants from the former group suffered from DM for more than ten years (32%) and 11 participants from the latter group suffered from DM for more than ten years (22%). Comparisons were additionally made on the basis of treatment therapy employed by the participants in the two groups. It was seen that the use of insulin as treatment was employed for 56% of group A participants; whereas, the remaining 46.6% experienced OHA treatment. However, with respect to patients from both groups who were treated through the combination of OHA and insulin, no notable difference was seen.

Of the 50 participants in group A, it was seen that six patients suffered from lesions and ulcerations situated on the leg, seven patients were afflicted with ankle ulcers, eight patients had ulcers on the Achilles tendon, six patients experienced them on the dorsum, eight patients reported having ulcers at the interdigital region, six patients presented with ulcers on the plantar region, and lastly, nine patients were afflicted with toe ulcers (data not presented in table). Trends of smoking were noted across the three study groups, having a prevalence of 58%, 24%, and 12% in groups A, B, and C respectively. Additionally, group A reported the highest prevalence of hypertension at 56%. Moreover, it was seen that group A patients were afflicted with other DM-related conditions such as neuropathy, nephropathy, and retinopathy at a notably higher rate than patients belonging to group B. It was seen that the percentage of HbA1c was significantly higher in group A (8.4 ± 2.35%) as compared to group B (7.6 ± 1.21%) and C (5.4 ± 0.6%). Additionally, the BMI of group A was significantly higher (23.37 ± 3.45 kg/m^2^) as compared to group B (22.54 ± 3.11 kg/m^2^) and C (21.47 ± 1.13 kg/m^2^). Higher values were further noted in group A with respect to serum creatinine (1.12 ± 0.56 mg/dL) as compared to group B (1.02 ± 0.25 mg/dL) and C (0.79 ± 0.15 mg/dL). The total cholesterol was significantly higher in group B (167.54 ± 34.84 mg/dL) in comparison to group A (147.50 ± 14.89 mg/dL) and C (115.21 ± 22.12 mg/dL). Furthermore, LDL-C levels were higher in C (98.32 ± 27.30 mg/dL) as compared to group A (81.20 ± 17.26 mg/dL) and B (97.32 mg/dL). The HDL-C values was lower in group A (35.21 ± 4.21 mg/dL) as compared to group B (40.89 ± 4.74 mg/dL) and C (54.28 ± 5.14 mg/dL). The level of triglycerides in group B was higher (132.01 ± 11.42 mg/dL) than group A (95.29 ± 17.76 mg/dL) and C (91.24 ± 25.57 mg/dL) (Table 1).

Table 2 has demonstrated the different concentrations of HSP70 in the three study groups. It was seen that group A presented with a significantly higher concentration of mean circulating plasma HSP70 (3319.82 pg/mL) as compared to B (2053.65 pg/mL) and C (1453.63 pg/mL).

Furthermore, Table 3 indicates the results from the multivariate analysis on the basis of odds ratio (OR), risk ratio (RR) and Chi Square test (*X*^2^). It was seen that a few variables showed a significantly positive relationship with the development of ulcers. These were inclusive of BMI (>25 kg/mt^2^) (OR 1.63, RR 1.25, *X*^2^ 24.14, *p* < 0.001), hypertension (OR 1.27, RR 1.06, *X*^2^ 5.01, *p* = 0.04), neuropathy (OR 4.75, RR 4.23 *X*^2^ 41.1, *p* < 0.0001), smoking (OR 5.21, RR 3.01, *X*^2^ 36.28, *p* < 0.0001), HbA1c (>6.9%) (OR 4.01, RR 1.76, *X*^2^ 21.13, *p* < 0.001), HDL-C (<40 mg/dL) (OR 1.54, RR 1.18, *X*^2^ 0.31, *p* < 0.004), retinopathy (OR 3.12, RR 1.77, *X*^2^ 21.4, *p* < 0.0001), and nephropathy (OR 3.44, RR 1.82, *X*^2^ 18.23, *p* < 0.001).

Additionally, the genotypic and allelic frequencies of the HSP70-hom gene (SNP) across the three groups has been presented in Table 4. It was seen that the C/T genotypic frequency was higher in group B (56%) as compared to A (54%) and C (50%). C/C genotypic frequency presented with the highest percentage in group C (36%) in comparison with B (30%) and A (10%). With respect to the T/T genotype frequency, it was seen that this was higher in group A (76%) in comparison to B (44%) and C (14%). The T allele frequency was higher in group A (7.3%) as compared to B (5.5%) and C (3.9%). This may be contrasted with the C allele frequency, which was noted to be highest in group C (6.1%) as compared to B (4.4%) and A (2.6%).

Table 5 presents the multivariate analysis of the DNA results. Specifically, the potential relationship between the HSP70-hom alleles and genes and groups A, B, and C was investigated. As highlighted earlier, group A was composed of DFU-afflicted patients, group B comprised diabetic subjects, and group C was the control group. As indicated by the results, the T/T homozygous frequency of the HSP70-hom presented with a significantly positive relative risk in group A, rather than groups B and C. Specifically, the homozygous frequency presented OR and RR with a 19-fold increase and 5-fold increase respectively in group A when compared to group B (OR 19.45; RR 5.42; *X*^2^ 38.8, *p* < 0.0001). The OR and RR of group A was 4-fold and 1.75-fold respectively in comparison to group C (OR 4.03(1.71–9.48); RR 1.72(1.21–2.44); *X*^2^ 10.6, *p* < 0.001). It is additionally important to note that a significantly negative relationship was seen under the condition that the C/C homozygous frequency of HSP70-hom in group A was almost 81% with a 75% risk reduction in OR, in addition to a 73% and 67% risk reduction as compared to group B (OR 0.19(0.06–0.58); RR 0.27(0.11–0.69); *X*^2^ 9.54, *p* < 0.002) and group C (OR 0.25(0.08–0.78); RR 0.33(0.13–0.84); *X*^2^ 6.24, *p* < 0.001), respectively.

Furthermore, the C/T heterozygous frequency of HSP70-hom experienced a decline among participants of group A, showing an absolute negative risk in comparison to group B (100%) and group C (90%). These results were similar to those obtained for RR in group A as compared to group B and C. Furthermore, the 2.2-fold and 5.4-fold rise of risk development in group A was estimated against group B and group C respectively in their total allelic frequency of HSP70-hom T allele in a homozygous or heterozygous state. These results were similar to the results obtained when using the Chi-square test (*X*^2^ 9.78, *p* < 0.001) for patients belonging to group A in comparison to groups B and C (*X*^2^ 36.1, *p* < 0.0001). As per the results obtained, a relationship was found between polymorphism of HSP70-hom and the foot ulcers experienced by diabetic patients, which was only restricted to T allele, whether in homozygous or heterozygous states. Whereas, it was seen that the homozygous C allele condition is a protective allele, which is independent of foot ulcers, as seen in North Indian diabetic populations.

Table 6 indicates the surgical procedures undertaken by the patients in accordance with the HSP70 genotype. It was seen that 26% of the patients belonging to group A having had either a major or minor amputation during the treatment, possessed a HSP70-hom T/T genotype. This is a greater value than seen for patients exhibiting the HSP70-hom C/T and HSP70-hom C/C genotype (14%). Likewise, it was seen that patients who experienced debridement of infection in their foot formed 14% of the group A population and had the HSP70-hom T/T genotype. This was followed by patients having the HSP70-hom C/T (10%) and HSP70-hom C/C genotype (6%).

## 4. Discussion

This study had aimed to investigate the potential significance of HSP70-hom gene SNP with respect to ulcer severity in diabetic patients. As had been highlighted earlier, DM sees an increasing global prevalence and is significantly associated with comorbidities that greatly reduce an individual’s quality of life [19]. More to the context of this study, it was highlighted that patients afflicted with DM have a higher probability of experiencing DFU, which has a high cost of management [20]. The present study noted that there was a higher predominance of male diabetic patients experiencing DFU as compared to female ones, which was in line with previous literature [21]. It was indicated that the causes for male preponderance may be linked to activity levels, hormonal variations, smoking trends, denial level, degree of compliance in addition to quality of education [22]. This was similar to the results obtained by this study, where it was seen that smoking was a significant risk factor in foot ulcer development.

It was seen that the average age of the participants mostly lay between 41 to 60 years, which is similar to the demographics reported by previous studies in this regard [23,24]. The present study reported the results of participants suffering from diabetes for a long duration of time, which was consistent with previous literature [25,26,27]. The development of foot ulcers is closely linked to the presence of ischaemia and neuropathy, which denote two significant complications of DM [28]. Patients from poor socioeconomic backgrounds, exhibiting poor knowledge regarding appropriate foot care and the importance of foot examination, are placed at added risk for the development of foot ulcers [29].

It was highlighted earlier that smoking was found to be a significant risk factor for foot ulcer development. Additionally, nephropathy and retinopathy were significant risk factors contributing to this condition, a finding which was similar to those reported by previous studies undertaken by the researchers [4,7]. The present study further reported that diabetic patients experiencing foot ulcer possessed low levels of HDL-C and LDL-C as seen in diabetic patients with no DFU, which was similar to previous literature [30]. The presence of lipids serves as a major risk factor for DFU development, as indicated by [31].

Relevant to the context of this study was the concept of hsps, which are synthesized under conditions of high stress and shown to be extremely significant in diabetic foot ulcer treatment [32]. As indicated by this study, the diabetic patients experiencing DFU exhibited high plasma concentrations of HSP70. This may be explained by previous studies indicating the abilities of hsps to act as antioxidants and improve the diabetic immune mechanism [33]. Hsps are shown to greatly expedite the wound-healing process due to their ability to neutralize protein denaturation within cells and tissues [8]. Furthermore, the polymorphism of the Ncol HSP70-hom gene was closely linked to diabetes, as indicated by Dhamodharan et al. [34].

As indicated by the present study, the HSP70-hom T/T genotype was significantly associated with high OR and RR for foot ulcer in diabetic subjects as compared to diabetic subjects without foot complications (who were admitted to the hospital for other causes) and healthy controls. Furthermore, the results of this study indicated that the presence of the T/T genotype was significantly increased in DFU. This was in line with previous studies [9]. However, a distinction may be established between those studies and this present one. For instance, the study by Mir et al. [18] suggested that the HSPA1B AG genotype was significantly associated with more severe local disease; amputation and a longer hospital stay than the GG genotype in DFU patients and this study elucidates the result without a healthy control group. Furthermore, the study conducted by [9] compared the genotypes of DFU with healthy controls.

HSP A1B 1267 polymorphism in hepatocellular carcinoma (HCC) is a silent mutation, which works as a surrogate genetic marker for risk of HCC [35]. P2/P2 polymorphism of HSP70-2 is a significant factor for accessing the low-risk of gastric cancer in females [36]. Liver failure or one factor of complications documented by Denver multiple organ failure scores was associated with HSPA1L genotype CT [37]. There were only a few reports of HSP70-hom polymorphism, substitution of Met-Thr amino acid at 493 positions was associated with T1DM [38], spondyloarthropathies [39], and sarcoidosis [40]. According to best of my knowledge, only a few studies have been published on the possible association of HSP70-hom polymorphism.

In contrast to the above methodology, this study made a comparison between the genotype results obtained for diabetic patients experiencing DFU and diabetic subjects without foot ulcer in addition to healthy controls. Therefore, a more wholesome understanding is gained of the close association between the role of HSP gene polymorphism and diabetic foot ulcers.

## 5. Conclusions

The study has aimed to investigate the close association between HSP70 polymorphism and the treatment of diabetic foot ulcers. The findings indicated that T/T genotype is significantly and positively associated with predispositions to foot ulcerations among patients, suffering from T2DM in North Indian populations. The novelty of the present study was seen through recruiting patients that were similar in factors such as age, sex, and ethnic region. The findings further indicated that there was a higher plasma concentration of HSP70 in diabetic patients suffering from DFU. However, the gene expression pertinent to the context of this study was not examined. Therefore, further studies are recommended to be carried out with respect to different ethnic regions to gain a more wholesome understanding of HSP polymorphism in diabetic patients exhibiting DFU.

## Figures and Tables

**Table 1:** General and demographic variables in cases and controls.

	Group A	Group B	Group C	*p*
Factors	Diabetic Patients	Diabetic Patients	Healthy Control	Value
	with Ulcer (*N* = 50)	without Ulcer (*N* = 50)	(*N* = 50)	<0.05
Age (years)	48.70 ± 9.34	48.79 ± 10.06	47.89 ± 9.56	No
Male/Female	33(66.0)/17(34.0)	31(62.0)/19(38.0)	29(58.0)/21(42.0)	No
Smoking (Yes/No)	29(58.0)/21(42.0)	12(24.0)/38(76.0)	6(12.0)/44(88.0)	Yes
Diabetes duration > 10 year	16(32.0)	11(22.0)	-	Yes
BMI (kg/sq mt)	23.37 ± 3.45	22.54 ± 3.11	21.47 ± 2.13	No
Systolic BP (mmHg)	141.13 ± 16.38	124.23 ± 14.87	115.87 ± 6.32	Yes
HbA1c (%)	8.4 ± 2.35	7.6 ± 1.21	5.4 ± 0.6	Yes
Fasting BG (mg/dL)	154.23 ± 46.58	141.20 ± 33.12	88.12 ± 16.46	Yes
Postprandial BG (mg/dL)	192.54 ± 41.76	198.32 ± 45.59	134.25 ± 34.21	Yes
Serum creatinine (mg/dL)	1.12 ± 0.56	1.02 ± 0.25	0.79 ± 0.15	Yes
LDL-C (mg/dL)	81.20 ± 17.26	97.32 ± 35.64	98.32 ± 27.30	Yes
HDL-C (mg/dL)	35.21 ± 4.21	40.89 ± 4.74	54.28 ± 5.14	Yes
Total cholesterol (mg/dL)	147.50 ± 14.89	167.54 ± 34.84	115.21 ± 22.12	Yes
Triglycerides (mg/dL)	95.29 ± 17.76	132.01 ± 117.42	91.24 ± 25.57	Yes
Neuropathy	34(68.0)	7(14.0)	-	Yes
Retinopathy	35(70.0)	18(36.0)	-	Yes
Nephropathy	32(64.0)	13(26.0)	-	Yes
Hypertension	28(56.0)	19(38.0)	-	Yes
CAD	18(36.0)	4(8.0)	-	Yes
PAD	31(62.0)	9(18.0)	-	Yes
Therapy			-	
Insulin	22(56.0)	8(30.0)	-	Yes
OHA	12(26.66)	24(46.6)	-	Yes
Both	16(20.00)	18(23.3)	-	No
Grade of ulcer (Texas)			-	
1	11(22.0)	-	-	-
2	32(64.0)	-	-	-
3	7(14.0)	-	-	-

BMI: Body mass index, Systolic BP: Systolic blood pressure; Fasting BG: fasting blood glucose; Postprandial BG: Postprandial blood glucose; CAD: coronary artery disease; PAD: Peripheral artery disease; OHA: Oral Hypoglycaemic Agents; HDL-C: High-density lipoproteins; LDL-C: Low-density lipoprotein. Categorical data were compared between the all groups using the *X*^2^ test. Normally distributed data were tested using one-way ANOVA. Non-normally distributed data were first analyzed using the Kruskal Wallis test, followed by the Mann-Whitney U test between all pair combinations of the group A; diabetic patients with foot ulcer., group B; diabetic patient without foot ulcer and group C; healthy control.

**Table 2:** Heat Shock Protein 70 Concentrations in Group A, Group B, and Group C Patients.

Heat Shock Protein 70 (pg/mL)		**Group A**	**Group B**	**Group C**	***p* Value**
	mean ± SD	3319.82 ± 1570.33	2053.65 ± 9454.24	1453.63 ± 944.24	<0.001
	CI of mean	586.37	356.87	352.58	
	Median	3229.01	1625.71	1025.71	
	(25–5%)	(1984.50–4137.16)	(1435.11–2253.58)	(835.11–1653.58)	

**Table 3:** Chi Square Test, Odds Ratio, and Risk Ratio to Study the Independent Variable Predicting Foot Ulcer in Diabetic Patients.

Independent Variable	Odds Ratio	Risk Ratio		*X*^2^ Test
	OR(95%CI)	RR(95%CI)	*X* ^2^	*p*
BMI (>25 kg/mt^2^)	1.63(1.45–2.96)	1.25(1.24–1.68)	24.14	<0.001
HbA1c (>6.9%)	4.01(1.37–7.29)	1.76(1.46–2.15)	21.13	<0.001
Total cholesterol (>150 mg/dL)	0.17(0.23–0.45)	0.32(0.14–0.45)	64.41	<0.001
Triglycerides (>200 mg/dL)	1.07(0.69–1.67)	1.02(0.83–1.29)	2.04	0.316
HDL-C (<40 mg/dL)	1.54(1.15–2.74)	1.18(1.02–1.59)	0.31	0.0045
LDL-C (>100 mg/dL)	1.46(0.86–2.28)	1.17(0.91–1.54)	0.06	0.954
Neuropathy	4.75(3.67–9.6)	4.23(2.30–3.97)	41.44	<0.0001
Retinopathy	3.12(1.91–5.11)	1.77(1.38–2.53)	21.45	<0.0001
Hypertension	1.27(0.71–1.87)	1.06(0.84–1.38)	5.01	0.04
Nephropathy	3.44(2.17–5.78)	1.82(1.44–2.55)	18.23	<0.0001
Smoking	5.213(3.02–6.86)	3.01(1.71–3.11)	36.28	<0.0001

The following independent variables were considered for the model: BMI: Body mass index; HbA1c (>6.9%); Total cholesterol; HDL-C: High-density lipoproteins; LDL-C: Low-density lipoprotein; Neuropathy; Retinopathy; Hypertension; Smoking. Only the variable that had a *p* value < 0.05 were considered in the final fitted model. OR = Odds ratio, RR= Risk ratio, CI= confidence interval, *X*^2^ = chi-square.

**Table 4:** Genotypic and Allelic frequencies of HSP70 hom gene single nucleotide polymorphism (SNP) in study groups.

Genotypic Frequencies	Group A (*n* = 50)	Group B (*n* = 50)	Group C (*n* = 50)
HSP7—hom C/C	5 (10.0)	15 (30.0)	18 (36.0)
HSP7—hom C/T	27 (54.0)	28 (56.0)	25 (50.0)
HSP7—hom T/T	38 (76.0)	22 (44.0)	7 (14.0)
Allelic frequencies	Group A (*n* = 50)	Group B (*n* = 50)	Group C (*n* = 50)
C	37(2.6)	58(4.4)	61(6.1)
T	103(7.3)	72(5.5)	39(3.9)

**Table 5:** Association of HSP70 hom gene polymorphism with DFU: ORs and RRs for major alleles and their homozygous and heterozygous genotypes.

SNP	Odds Ratio (95% Ci)	Risk Ratio (95% Ci)	*X* ^2^	*p*
Group A compared with Group C				
C/C	0.25[0.08–0.78]	0.33[0.13–0.84]	6.25	0.001
C/T	0.98[0.41–2.02]	0.96[0.67–1.37]	0.004	0.84
T/T	4.03[1.71–9.48]	1.72[1.21–2.44]	10.67	0.001
Group A compared with Group B				
C/C	0.19[0.06–0.58]	0.27[0.11–0.69]	9.54	<0.002
C/T	1.17[0.53–2.57]	1.08[0.74–1.57]	0.16	0.68
T/T	19.45[6.45–54.44]	5.42[2.68–10.98]	38.83	<0.0001
Group A compared with Group C				
C	0.18[0.10–0.32]	0.39[0.29–0.54]		
T	5.4[3.08–9.71]	2.18[0.61–2.95]	36.18	<0.0001
Group A compared with Group B				
C	0.44[0.26–0.74]	0.59[0.42–0.82]		
T	2.24[1.34–3.73]	1.32[1.10–1.59]	9.78	0.001

OR = Odds ratio, RR = Risk ratio, CI = confidence interval, *X*^2^ = chi-square. The Chi-square test was used whether significant difference (*p*-value) in genotype frequencies were observed when groups was compared.

**Table 6:** Extent of interventions of genotype in relation to diabetic foot ulceration treatment.

Treatment/Genotype	Conservative 9(18.0)	Debridement 15(30.0)	Amputation	
			Minor 20(40.0)	Major 6(12.0)
HSP7—hom C/C	2	3	1	0
HSP7—hom C/T	4	5	6	1
HSP7—hom T/T	3	7	13	5

Minor: trans metatarsal, Major: Below/above knee amputations.

## Availability of Data and Material

The data were available in the record section of the Rajiv Gandhi Centre for Diabetes and Endocrinology, Aligarh Muslim University, Aligarh INDIA.
